# Exploring changes, and factors associated with changes, in behavioural determinants from a low-cost, scalable education intervention about knee osteoarthritis: An observational cohort study

**DOI:** 10.1186/s12891-021-04751-2

**Published:** 2021-10-09

**Authors:** Thorlene Egerton, Joanne Bolton, Camille E. Short, Kim L. Bennell

**Affiliations:** 1 Centre for Health, Exercise and Sports Medicine, The University of Melbourne, Level 7, Alan Gilbert Building, Melbourne, Victoria 3010 Australia; 2 Physiotherapy Department, The University of Melbourne, Melbourne, Australia; 3 Melbourne Centre for Behaviour Change, Melbourne School of Psychological Sciences, The University of Melbourne, Melbourne, Australia; 4 Melbourne School of Health Sciences, The University of Melbourne, Melbourne, Australia

**Keywords:** Patient education, Knee, Osteoarthritis, Self-management, Self-efficacy, Health literacy

## Abstract

**Objective:**

To explore the relationships between participant characteristics, perceptions of a short educational video about osteoarthritis and its management, and immediate changes in behavioural determinants for effective self-management behaviours.

**Methods:**

Seventy-eight participants with knee OA (77% female, mean age 63.0 ± 8.7) watched the 9-min video that included evidence-based content and was designed to foster empowerment to self-manage effectively. Data were collected by online questionnaire at baseline and immediately after watching the video. Associations were tested between baseline health and information processing characteristics (health literacy, need for cognition), perceptions of the video (enjoyment, helpfulness, believability, novelty and relevance) and pre-post changes in behavioural determinants (self-efficacy for managing arthritis, attitude to self-management or ‘activation’, and importance/confidence for physical activity).

**Results:**

All behavioural determinants improved immediately after watching the video. Positive perceptions were associated with greater improvements in self-efficacy for arthritis (Spearman’s rho, *ρ* = 0.26–0.47). Greater perceived relevance was associated with increased self-rated importance of being physically active (*ρ* = 0.43). There were small positive associations between health literacy domains related to health information and positive viewer perceptions of the video. People with higher need for cognition may achieve greater improvement in confidence to be physically active (*ρ* = 0.27).

**Conclusion:**

The educational video may help achieve outcomes important for increasing self-management behaviours in people with knee osteoarthritis. Positive perceptions appear to be important in achieving these improvements. People with lower health literacy and lower need for cognition may respond less well to this information about knee osteoarthritis delivered in this way.

**Supplementary Information:**

The online version contains supplementary material available at 10.1186/s12891-021-04751-2.

## Introduction

Patient education is the most commonly used intervention for chronic disease management [1]. Education interventions have variably been shown to have benefits for chronic disease management in general and musculoskeletal pain disorders more specifically [1–5]. Although impacts are modest, education interventions are accepted as an important part of multi-component behaviour change interventions. Knee osteoarthritis (OA) is a highly prevalent, chronic painful condition with no cure, and recommended care focusses on education and self-management behaviours. Education is recommended as a core component of knee OA management [6–8], yet not all education is beneficial – it can do harm via the nocebo effect whereby expectation of symptom worsening is unintentionally promoted [9]. People who are fearful of activity because of their understanding of their joint damage are less likely to engage in the very treatment that can alleviate symptoms and preserve joint functioning [10]. Education for people knee OA has received little research attention and little is known about the comparative impacts of different types of education [6].

The potential benefits of education interventions for knee OA include improvements in psychosocial outcomes [2–4], pain [4, 5], function [4, 11, 12], and markers of disease [1, 5]. To achieve these benefits, education should not just share information about the disease process but develop a patient mindset and level of understanding that facilitates ongoing physical activity, participation and well-being [13]. Education should aim to activate the person to self-manage through changes in behavioural determinants such as expectations, motivation and self-efficacy [14–18]. Traditional patient education for people with knee OA focuses on structural damage (e.g. cartilage degeneration or ‘wear and tear’) and an expectation of disease progression (e.g. symptoms will progress and surgery is inevitable) [19]. This type of information is typical of education currently delivered by healthcare professionals [20, 21] as well as via written and online resources. This information has been shown to foster negative outcome expectation, fatalism and activity avoidance in people with knee OA [13, 20, 22, 23]. Previous research suggests that patient education for people with musculoskeletal pain delivered with a biopsychosocial approach and messages of empowerment and positive expectation of benefit from conservative options, may be more beneficial than traditional disease information approaches [3, 24, 25]. Our own qualitative study exploring the reactions of people with painful knee OA to a brief educational video with novel empowerment content including psychosocial components of the condition, found most participants responded favourably [26]. Many declared an intention to add at least one effective self-management behaviour. However, there was a range of responses and a small proportion reported less favourable reactions to the video. These included frustration (e.g., because the information was not what they wanted to hear) and some resistance either because the information was at odds with their beliefs or advice from their doctor, or because they perceived the recommendations did not apply to their personal situation [26]. This variability in responses warrants further exploration in order to optimise utilisation of low-cost, scalable education and target enhanced education approaches.

**Fig. 1:**
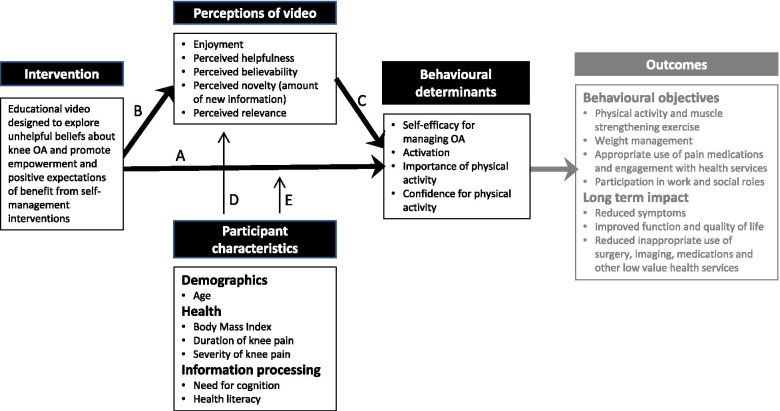
Intervention logic of the educational intervention designed to foster self-management behaviours. The intervention is theorized to impact on behavioural determinants directly (**A**) and indirectly via positive perceptions of the video (**B** and **C**). Participant characteristics are possible predictors of perceptions of the video (**D**) and of the impact of the video on behavioural determinants (**E**). Outcomes were not measured in this study

This quantitative study aimed to further explore responses to this educational video. The video utilises presentation features and content that moves away from the more common biomedically based-education and pathoanatomical approaches to explaining and managing the disease. It avoids pictures of structural damage and does not describe severity in terms of imaging results. It was designed to communicate positive expectations about the effects of self-management behaviours with optimism for one’s future prognosis [16]. Our rationale for how the educational video would achieve benefits for people with knee OA is depicted in the proposed intervention logic (Fig. 1).

The study had three objectives. Firstly, to investigate whether the behavioural determinants targeted by the intervention (self-efficacy for managing OA, ‘activation’ i.e. attitude towards self-management, importance of physical activity, and confidence to be sufficiently physically active) changed immediately after watching the video (analysis A). The second objective was to describe viewer perceptions of the video (enjoyment, helpfulness, believability, novelty and relevance) (analysis B), and then explore whether viewer perceptions were associated with changes in behavioural determinants (analysis C). The third objective was to identify participant characteristics (age, health status and information usage attributes, i.e. health literacy and need for cognition) that were associated with viewer perceptions (analyses D) and/or changes in behavioural determinants (analysis E).

### Significance

Successful adult patient education is a planned intervention, grounded in adult learning theory, with multiple components that need careful consideration [27]. Exploring potential causal pathways explaining the effect of this novel educational intervention may help to identify characteristics that are important for educational interventions for this patient population. Since people function in complex social and physical environments and bring a range of personal attributes and health beliefs to their self-management of any health condition, understanding the participant characteristics that may indicate who will/will not benefit from this low cost, scalable education intervention may help with planning more tailored implementation strategies and optimise utilisation.

## Methods

This exploratory study utilised data collected as part of an evaluation of a novel patient education video [26]. The study was approved by the School of Health Sciences Human Ethics (University of Melbourne: ID 1953681) and the report adheres to the STROBE Statement [28]. All participants provided informed consent. Further methods details can also be found in the published qualitative study [26].

### Intervention

The 9-min knee OA info video “Knee Pain: What can I do?” [29] was created with consideration of both content (factual, meeting people’s information needs) and delivery (engagement, education pedagogy) [26]. The content reflects contemporary understanding of pain neurobiology and optimal OA management i.e. moving away from a biomedical focus on structural damage and reliance on medication and surgery, towards a biopsychosocial and self-management approach to long-term management. Some features of the design were a deliberate attempt to help avoid creating resistance to some of the content that may challenge some people’s beliefs and identity. The design was also based on theories of behaviour change (Social-cognitive theory [30] and the Information-Motivation-Behavioural skills model [31]), included several behaviour change techniques (chiefly cognitive restructuring, information about health and social consequences, verbal persuasion about capability, social support, and credibility of source [32]), and targeted known barriers to effective self-management behaviours such as focussing attention on structural changes and being fearful of doing further damage [33]. In terms of adult education pedagogy, the video was based on a constructivist approach to learning whereby viewers are active participants in new knowledge creation. In that respect, the video validates concerns about living with the disease, and viewers are guided to reflect on their own experiences via open ended questions to create cognitive dissonance in relation to unhelpful beliefs about knee OA [13]. The goal of the short video was to create a positive mindset using contemporary information about knee OA delivered in a way that empowers people to seek further information on how they can actively engage in managing their condition.

### Participants and recruitment

One hundred Australian participants were recruited via social media, community advertisement and word of mouth during June–November 2019. Participants were eligible if they had knee OA based on self-reported physician diagnosis or clinical diagnostic criteria [7]. Exclusion criteria included: inflammatory arthritis; arthroplasty (any joint), prior or current participation in care with a focus on self-management education e.g. chronic pain program or tertiary OA program; or difficulty communicating in English. A sample size of 100 participants would give a 95% confidence interval margin of error of about 0.2 standard deviations around a sample mean for outcome measures (i.e. a confidence interval of width of 0.4 standard deviations). This was considered appropriate for an exploratory study.

### Data collection

**Fig. 2:**
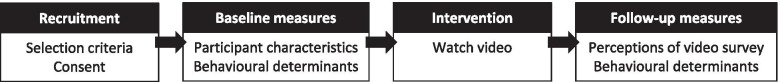
Study timeline

Participants completed a baseline questionnaire including demographic details (age), health factors (anthropometry, knee pain history), and information usage attributes (health literacy, need for cognition). The questionnaire also included behavioural determinants that were hypothesised to be important mechanisms for the intervention to be effective in facilitating better self-management (self-efficacy for managing OA, activation, and importance and confidence to be physically active) [15, 30, 31, 34–37]. After completion of the baseline questionnaire, participants were sent the link to the video and asked to repeat the behavioural determinants questionnaire plus a custom-made survey to determine viewer perceptions of the video immediately afterwards. Study timeline shown in Fig. 2. Measurement tools/survey items are briefly described below with further detail provided in Additional file 1.

### Participant characteristics

Pain (average over past week and worst pain during activity) was self-reported using an 11-point numerical rating scales (NRS) [38]. Health literacy was measured using the 44-item Health Literacy Questionnaire [39], a comprehensive measure of nine domains of health literacy with established validity. Four of the health literacy domains are related to health information: ‘*Having sufficient information to manage my health*’, ‘*Appraisal of health information*’, ‘*Ability to find good health information*’ and ‘*Understand health information well enough to know what to do*’. ‘*Need for Cognition*’, a personality trait relating to how people tend to process information, was measured using three items with 11-point NRS [40]. People who process information with minimal effort and using short cuts (e.g., heuristics) are said to have lower need for cognition [41]. They often decide whether to believe information based on the source of the information or emotional responses. However, people with higher need for cognition have a preference for thinking actively and critically when processing information [41]. Along with health literacy, need for cognition may influence how an individual responds to the video and if they benefit from watching it [40]. To be effective, the video should engage all people regardless of their health literacy, need for cognition and other individual characteristics.

### Viewer perceptions of the video

The survey included questions on perceived enjoyment, helpfulness, believability (degree to which the information was believed to be true), novelty (amount of new information), and relevance of the information (average of three items); all rated on 0–6 Likert scales [26]. Scores less than 4/6 for enjoyment, helpfulness, believability and relevance were considered negative responses, while a score of less than 2/6 was considered negative for amount of new information.

### Behavioural determinants

Activation (or attitude towards self-management) was measured with the 13-item Patient Activation Measure (PAM) [11, 42]. Self-efficacy for managing OA was measured using the Arthritis Self-Efficacy Scale (ASES) [43]. The ASES has three subscales: Pain, Function and Other Symptoms. Only three of the nine items from the function subscale were measured as the other items were not relevant to knee OA. Importance of physical activity and confidence to be sufficiently physically active were each rated on an 11-point NRS [31].

### Analysis

Baseline data are described using mean (standard deviation, SD) and range. Pre-post changes in self-efficacy for managing OA, activation, and importance and confidence for physical activity were analysed using paired t-tests (analysis A). Viewer perceptions of the video (analysis B) are described using mean (SD), range and the proportion of participants with negative perceptions. To identify the perceptions of the video that were associated with changes in behavioural determinants (analysis C), Spearman’s rho correlation coefficient with 95% confidence intervals (CI) [44] were used. Participant characteristics that were associated with perceptions of the video (analysis D) and changes in behavioural determinants (analysis E) were identified using Spearman’s correlation with 95% confidence intervals (CI) [44]. Participant characteristics were age, BMI, severity of knee pain, need for cognition, and the four health information domains of health literacy (Fig. 1). Interpretation of correlation analyses was guided by the criteria 0 - < 0.32 = questionable, ≥0.32 - < 0.40 = fair, and ≥ 0.40 = moderate. The cut off of 0.32 was selected as the lower limit because the 95%CI lower bound for a Spearman’s correlation of 0.32 for *n* = 78 is 0.10 [45–47], therefore a correlation of below 0.32 had a > 5% chance of actually being negligible. Analyses were carried out with SPSS (IBM Corp SPSS Statistics for Windows, Version 26.0. Armonk, NY).

## Results

**Fig. 3:**
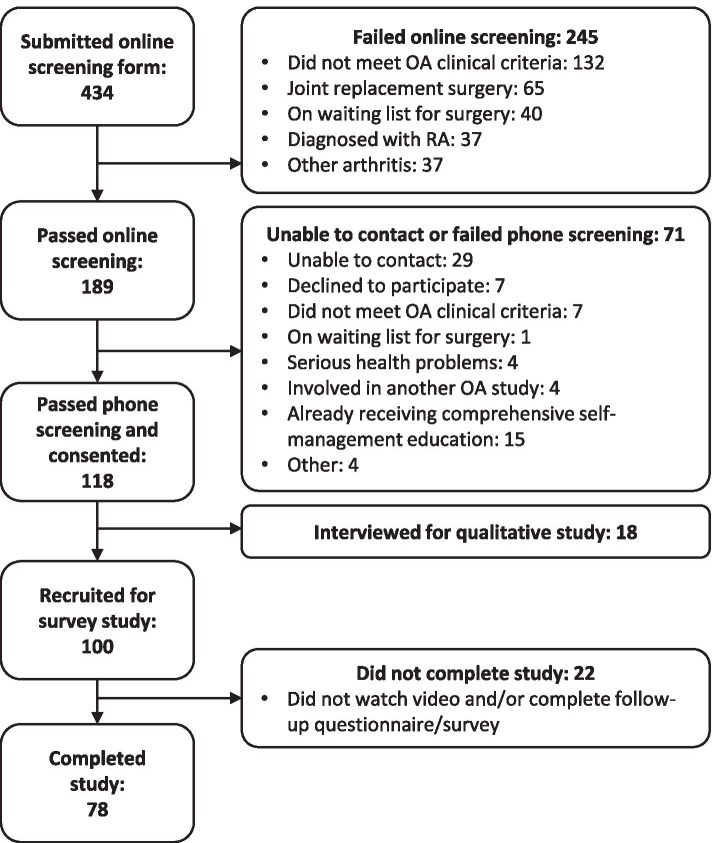
Flow diagram

Seventy-eight (78%) participants completed the study (Fig. 3). Non-completers (participants who did not submit the follow-up questionnaire and survey) were of similar sex, age, and BMI, but fewer non-completers had tertiary education and pain severity was higher for the non-completers. Participant characteristics are provided in Table 1. The sample included mostly females with an average age of 63 years and average BMI of about 30 kg/m^2^. The sample included a wide range of knee OA presentations in terms of pain severity and duration. Average health literacy was high (Table 1), and as a group there was a higher need for cognition (mean 7.5/10, SD2.1).

**Table 1:** Characteristics of participants

Characteristic	(***n*** = 78)
**Age, years** (average ± SD, range)	63.0 ± 8.7, 46–83
**Female** (n, %)	60 (77%)
**BMI, kg/m**^**2**^ (average ± SD, range)	29.8 ± 6.4, 20.7–55.3
**Painful knee(s)** (n, %)	
Left knee	18 (23.1%)
Right knee	19 (24.4%)
Both knees	41 (52.6%)
**Duration of pain in worst knee** (n, %)	
Less than 1 year	8 (10.3%)
1 or 2 years	12 (15.4%)
3 to 5 years	23 (29.5%)
5 to 10 years	19 (24.4%)
More than 10 years	16 (20.5%)
**Pain,** NRS 0–10 (average ± SD, range)	
Average pain over past week in most painful knee?	4.6 ± 1.9, 0–8
Worst pain felt during activity over past week in most painful knee?	6.3 ± 1.8, 2–9
**Health Literacy Questionnaire** (average ± SD)^a^	
Feeling understood and supported by healthcare providers (0–4)	3.0 ± 0.5
Having sufficient information to manage my health (0–4)	2.8 ± 0.4
Actively managing my health (0–4)	3.0 ± 0.4
Social support for health (0–4)	2.9 ± 0.4
Appraisal of health information (0–4)	3.0 ± 0.3
Ability to actively engage with healthcare providers (0–5)	3.7 ± 0.6
Navigating the healthcare system (0–5)	3.6 ± 0.5
Ability to find good health information (0–5)	3.9 ± 0.5
Understand health information well enough to know what to do (0–5)	4.1 ± 0.4
**Need for Cognition** NRS 0–10 (average ± SD, range)^b^	
Average score of 3 items, 0–10	7.5 ± 2.1, 2–10

*NRS* numerical rating scale

^a^higher score signifies better health literacy

^b^higher score signifies greater need for cognition (which is indicative of a tendency to enjoy effortful cognitive activities)

### Pre-post change in behavioural determinants (A)

Results for the pre-post (within-group) comparison showed statistically significantly improved ratings for all outcomes including arthritis self-efficacy, activation and ratings for importance/confidence with physical activity (Table 2).

**Table 2:** Results for pre-post (within-group) comparison (*n* = 78)

	Baseline (mean ± SD)	Follow-up (mean ± SD)	Change (mean ± SD, 95% confidence interval)
Arthritis Self-Efficacy Scale (ASES):^a^			
Pain subscale (5 items, 1–10)	5.8 ± 1.7	7.2 ± 1.8	1.4 ± 1.8, 1.0 to 1.8
Function subscale (3 items, 1–10)	6.4 ± 2.7	7.5 ± 2.4	1.1 ± 1.4, 0.8 to 1.4
Other symptoms subscale (6 items, 1–10)	6.2 ± 1.6	7.4 ± 1.6	1.3 ± 1.4, 0.9 to 1.6
Patient Activation Measure (PAM)^a^			
Average of 13 items (1–4)	3.3 ± 0.4	3.5 ± 0.4	0.23 ± 0.35, 0.16 to 0.31
Importance of regular physical activity (0–10)^a^	8.8 ± 1.5	9.3 ± 1.1	0.4 ± 1.3, 0.1 to 0.7
Confidence can achieve sufficient physical activity (0–10)^a^	6.6 ± 2.5	7.4 ± 2.1	0.8 ± 2.0, 0.4 to 1.3

^a^higher score indicates better/greater

### Viewer perceptions of the video (B)

Viewer perceptions of the video are detailed in Table 3. While most perceptions were positive, a small but notable number of people indicated they did not enjoy the video (10%), did not find it helpful (10%), were not convinced the information in the video was true and correct (4%), thought there was no or minimal new information in the video (23%), and/or did not think it was particularly relevant to them (14%).

**Table 3:** Viewer perceptions of the video with proportion reporting negative perceptions (*n* = 78)

Item	Score (average ± SD, range)	Number (%) with negative response
Enjoyability of the video (0 = not at all enjoyable – 6 = very enjoyable)	5.0 ± 1.0, 2–6	< 4/6: 8 (10%)
Degree to which the video was helpful (0 = not at all helpful – 6 = extremely helpful)	4.9 ± 1.2, 0–6	< 4/6: 8 (10%)
Believability (Belief that the information was true and correct) (0 = not at all – 6 = completely)	5.3 ± 0.8, 3–6	< 4/6: 3 (4%)
Perceived novelty (Amount of new information) (0 = no new information – 6 = a great deal of new information)	2.9 ± 1.7, 0–6	< 2/6: 18 (23%)
Perceived relevance (average of 3 questions, scored 0–6, higher = more relevant)	5.0 ± 1.0, 2–6	< 4/6: 11 (14%)

### Associations between perceptions of video and behavioural determinants (C)

Some viewer perceptions were associated with changes in the behavioural determinants (Table 4). Relevance was moderately positively associated with change in importance to be physically active (*ρ* = 0.41, 95%CI 0.20 to 0.59) such that the greater the perceived relevance, the greater the increase in ratings of importance of physical activity after viewing the video. Novelty (i.e. information in the video was perceived as being new) was positively associated with both change in self-efficacy for managing pain (fair association, *ρ* = 0.34, 95%CI 0.12 to 0.53) and change in self-efficacy for managing other symptoms (moderate association, *ρ* = 0.41, 95%CI 0.19 to 0.58). The more new information the participant perceived, the greater the increase in self-efficacy. Perceived helpfulness was also associated with increases in pain self-efficacy (fair, *ρ* = 0.32, 95%CI 0.10 to 0.51), and other symptoms self-efficacy (moderate, *ρ* = 0.47, 95%CI 0.26 to 0.63). Enjoyment was associated with change in ASES other symptoms (fair, *ρ* = 0.32, 95%CI 0.10 to 0.51), but the association between enjoyment and change in ASES pain was smaller and therefore considered questionable (*ρ* = 0.26, 95%CI 0.04 to 0.46). Viewer perceptions of believability did not seem to be associated with any changes to determinants. In addition, none of the perceptions measured in this study were associated with changes to activation or confidence to be physically active.

**Table 4:** Associations between viewer perceptions and pre-post change in behavioural determinants. Data are Spearman’s rho correlation coefficient with 95% confidence intervals, *n* = 78, bold signifies *ρ* ≥ 0.32

	Change in ASES (pain)	Change in ASES (function)	Change in ASES (other symptoms)	Change in PAM	Change in importance of physical activity	Change in confidence they can be sufficiently physically active
Enjoyable	0.26* (0.04 to 0.46)	0.10 (− 0.12 to 0.32)	0.32* (0.10 to 0.51)	0.02 (− 0.20 to 0.24)	0.20 (− 0.02 to 0.41)	0.06 (− 0.16 to 0.28)
Helpful	0.32* (0.10 to 0.51)	0.08 (− 0.15 to 0.29)	0.47* (0.26 to 0.63)	0.03 (− 0.20 to 0.25)	0.18 (− 0.04 to 0.39)	0.14 (− 0.08 to 0.35)
Believability	0.13 (− 0.09 to 0.35)	− 0.20 (− 0.40 to 0.03)	0.15 (− 0.08 to 0.36)	0.08 (− 0.15 to 0.30)	0.13 (− 0.10 to 0.34)	− 0.01 (− 0.23 to 0.21)
Novelty	0.34* (0.12 to 0.52)	0.05 (− 0.18 to 0.27)	0.41* (0.19 to 0.58)	0.13 (− 0.09 to 0.35)	0.23* (0.00 to 0.43)	0.10 (− 0.12 to 0.32)
Relevance	0.02 (− 0.20 to 0.25)	− 0.11 (− 0.32 to 0.12)	0.20 (− 0.03 to 0.40)	0.08 (− 0.15 to 0.29)	0.41* (0.20 to 0.59)	0.13 (− 0.09 to 0.35)

^*^p < 0.05

### Participant characteristics associated with viewer perceptions (D)

There were several questionable positive associations between domains of health literacy and the viewer perceptions of helpfulness and believability (Table 5), but the positive association between the health literacy domain of ‘Understand health information well enough to know what to do’ and perceived believability was considered fair (*ρ* = 0.36, 95%CI 0.14 to 0.54). These associations indicate better health literacy in relation to health information may lead to more positive perceptions of the video. Or conversely, that people with lower health literacy were more likely to react negatively. There was a questionable negative association between pain severity and believability (*ρ* = − 0.26, 95%CI − 0.46 to − 0.04). If true, that would mean, the more severe the pain, the less believable the information in the video.

**Table 5:** Participant characteristics associated with perceptions of the video. Data are Spearman’s rho correlation coefficient with 95% confidence intervals, *n* = 78 (apart from BMI where *n* = 77), bold signifies *ρ* ≥ 0.32

	Enjoyable	Helpful	Believability	Novelty	Relevance
Age	0.01 (− 0.21 to 0.23)	− 0.05 (− 0.27 to 0.17)	− 0.19 (− 0.40 to 0.04)	0.10 (− 0.13 to 0.32)	− 0.11 (− 0.33 to 0.12)
BMI	− 0.12 (− 0.34 to 0.11)	− 0.18 (− 0.39 to 0.05)	− 0.02 (− 0.24 to 0.20)	−0.05 (− 0.27 to 0.18)	0.03 (− 0.20 to 0.25)
Knee pain severity	0.12 (− 0.11 to 0.33)	0.05 (− 0.17 to 0.27)	−0.26* (− 0.46 to − 0.04)	0.01 (− 0.21 to 0.23)	−0.13 (− 0.34 to 0.10)
Need for cognition	− 0.05 (− 0.27 to 0.17)	−0.09 (− 0.31 to 0.14)	0.00 (− 0.22 to 0.22)	−0.12 (− 0.33 to 0.11)	−0.03 (− 0.25 to 0.19)
HL Having sufficient information to manage my health	0.09 (− 0.14 to 0.31)	0.16 (− 0.07 to 0.37)	0.14 (− 0.09 to 0.35)	−0.01 (− 0.23 to 0.21)	0.08 (− 0.15 to 0.30)
HL Appraisal of health information	0.19 (− 0.04 to −.40)	0.22* (0.00 to 0.42)	−0.03 (− 0.25 to 0.19)	−0.08 (− 0.30 to 0.15)	0.00 (− 0.22 to 0.22)
HL Ability to find good health information	0.17 (− 0.06 to 0.38)	0.19 (− 0.04 to 0.40)	0.28* (0.06 to 0.48)	0.10 (− 0.13 to 0.32)	0.14 (− 0.09 to 0.35)
HL Understand health information well enough to know what to do	0.23* (0.00 to 0.43)	0.27* (0.05 to 0.47)	0.36* (0.14 to 0.54)	0.13 (−0.10 to 0.34)	0.20 (− 0.03 to 0.41)

*HL* Health literacy

^*^p < 0.05

### Associations between participant characteristics and change in behavioural determinants (E)

There was a questionable positive association between need for cognition and change in confidence to be sufficiently active (*ρ* = 0.27, 95%CI 0.05 to 0.47) (Table 6). No other characteristics measured in this study were associated with changes in behavioural determinants.

**Table 6:** Participant characteristics associated with change in outcomes. Data are Spearman’s rho correlation coefficient with 95% confidence intervals, *n* = 78 (apart from BMI where *n* = 77), bold signifies *ρ* ≥ 0.32

	Change in ASES (pain)	Change in ASES (function)	Change in ASES (other symptoms)	Change in PAM	Change in motivation to be PA	Change in confidence to be sufficiently PA
Age	0.04 (− 0.18 to 0.26)	0.13 (− 0.10 to 0.34)	−0.06 (− 0.28 to 0.16)	−0.03 (− 0.25 to 0.19)	0.04 (− 0.18 to 0.26)	0.16 (− 0.07 to 0.37)
BMI	−0.12 (− 0.34 to 0.11)	0.08 (− 0.15 to 0.30)	−0.19 (− 0.40 to 0.04)	−0.01 (− 0.23 to 0.21)	0.14 (− 0.09 to 0.35)	0.07 (− 0.16 to 0.29)
Knee pain severity	−0.12 (− 0.33 to 0.11)	−0.11 (− 0.33 to 0.12)	−0.01 (− 0.23 to 0.21)	0.10 (− 0.13 to 0.32)	−0.19 (− 0.40 to 0.04)	0.03 (− 0.19 to 0.25)
Need for cognition	−0.01 (− 0.23 to 0.21)	−0.12 (− 0.33 to 0.11)	0.01 (− 0.21 to 0.23)	−0.02 (− 0.24 to 0.20)	0.11 (− 0.12 to 0.33)	0.27* (0.05 to 0.47)
HL Having sufficient information to manage my health	− 0.03 (− 0.25 to 0.19)	−0.19 (− 0.40 to 0.04)	−0.09 (− 0.31 to 0.14)	−0.08 (− 0.30 to 0.15)	−0.08 (− 0.30 to 0.15)	−0.14 (− 0.35 to 0.09)
HL Appraisal of health information	− 0.08 (− 0.30 to 0.15)	−0.11 (− 0.33 to 0.12)	−0.03 (− 0.25 to 0.19)	−0.05 (− 0.27 to 0.17)	−0.14 (− 0.35 to 0.09)	0.14 (− 0.09 to 0.35)
HL Ability to find good health information	0.08 (− 0.15 to 0.30)	−0.12 (− 0.33 to 0.11)	0.01 (− 0.21 to 0.23)	−0.08 (− 0.30 to 0.15)	−0.06 (− 0.28 to 0.16)	−0.00 (− 0.22 to 0.22)
HL Understand health information well enough to know what to do	0.01 (− 0.21 to 0.23)	−0.11 (− 0.33 to 0.12)	0.20 (− 0.03 to 0.41)	0.10 (− 0.13 to 0.32)	0.02 (− 0.20 to 0.24)	0.22 (− 0.01 to 0.42)

*ASES* Arthritis self-efficacy scale, *PAM* Patient Activation Measure, *PA* physical activity, *HL* Health literacy

^*^p < 0.05

## Discussion

This study showed that people report improvements in self-efficacy for managing their OA, activation, physical activity importance, and physical activity confidence immediately after watching a short educational video that delivers information about knee OA using a biopsychosocial explanation for the pain and a focus on empowerment for self-management. The perceptions of the video ranged amongst the participants with some rating the video negatively for enjoyment, helpfulness, believability, novelty and/or relevance. Positive responses to the video information were associated with greater improvements with self-efficacy for managing OA pain and other symptoms. Perceived greater relevance was associated with greater increase in rating of importance to be physically activity. Taken together the data supports a positive relationship between health literacy related to health information and how the video was received. There may be a negative association between pain severity and belief that the information was true and correct. Finally, there may be an association between greater need for cognition and reporting greater improvements in confidence to be physically activity immediately after watching the video.

Interpreting the clinical meaning of the changes in behavioural determinants is difficult as measurement error and minimal clinically important changes have not been determined for these measures. Comparing effect sizes (change score divided by baseline SD [48]) with comparable interventions may provide some insight into the importance of the findings. The effect sizes for within-group changes in arthritis self-efficacy domains of pain and other symptoms were greater than reported in other studies looking at education interventions for people with knee OA. The average within-group change effect size for ASES pain from five cohort studies and four RCTs on self-management education interventions was 0.39 and 0.37 respectively [17], compared with 0.82 for this video. Similarly, for ASES other symptoms, reported average within-group effect sizes were 0.28 (cohort) and 0.25 (RCTs), compared with 0.81 in this study. The effect size for PAM of 0.58 was comparable with within-group changes in PAM reported in other studies on people with knee OA testing interventions with a focus on supporting self-management (0.34 [49], 0.51 [50] and 0.57 [50]). The relatively large effect sizes for such a short and easily implementable intervention may alternatively be explained by the short period of time between intervention (viewing the video) and completing the follow up questionnaire.

The measures of importance/confidence to be physically active are measures of cognition rather than action but both are critical to motivation and thus to successful behavior change [31]. If a goal is not perceived to be ‘important’ or we do not believe we can achieve it, then we are unlikely to pursue it. It is difficult to say how high these scores need to be to translate into action because many factors affect action, e.g., stability of intention, control over time, social and environmental influences etc. The amount of change that is clinically important is not known for these scales. An increase of 0.4 NRS points for ‘importance’ may be clinically meaningful over a large population, however the lower bound of the 95%CI for change of 0.1 is unlikely to be clinically beneficial. ‘Confidence’ changed by 0.8 NRS points in our sample, which is more likely to be a clinically important impact of the education. The improvements in ASES and ‘confidence’ to be active may indicate the content and/or design/delivery features of this video were particularly helpful for improving self-efficacy. It must be emphasised that there was no comparator group in this study and these positive changes in all the measured behavioural determinants cannot necessarily be attributed to the intervention and may not be lasting.

Ratings of perceptions of the video were predominantly positive, however not everyone (10–23%) found the video enjoyable, helpful, felt there was new information, or perceived it to be relevant to them. It remains unclear whether any of the participant characteristics can predict more positive or negative perceptions, as most associations were of questionable strength. It is pleasing from the perspective of general applicability and acceptability of the video that strong associations were not apparent, however, there was a tendency for health literacy domains to be positively associated with perceptions, in particular helpfulness and believability. Most notably, self-reported ability to understand health information was associated with finding the video believable. Although most of these correlations were categorised as questionable based on our criteria, it does raise some concern that the video may be less useful for people who are potentially most vulnerable in terms of health literacy. Other studies have found that interventions benefitting people with higher health literacy also benefit those with lower [51] and this was our aim. Based on our findings we speculate that our contemporary description of knee OA as a whole-of-joint, chronic pain condition, influenced by psychological and social factors, may be more difficult to comprehend and may challenge previous information that explains OA as a simple problem of mechanical wear and tear for those with lower health literacy. Educational interventions for knee OA may be more effective for people with lower health literacy if delivered with additional support. The questionable negative association between severity of pain and belief that the information was true suggests that people with higher pain levels may be less likely to believe or trust the information. If this is true, people with severe pain may need different educational strategies or additional support to help them learn and understand about the benefits of lifestyle self-management interventions for them.

In line with our logic model, positive viewer perceptions were related to greater improvements in the behavioural determinants. Interestingly though, not all perceptions were correlated with changes in all determinants targeted. Relevance for example, was correlated only with change in rating of importance of being physically active. Whereas, novelty, helpfulness and finding the video enjoyable were associated with changes in arthritis self-efficacy. Based on extant frameworks and theorisations [52–54], it was assumed that all of these perceptions could lead to an increased attention towards, and acceptance of, the advice provided (i.e. increased persuasiveness), and therefore those with more positive perceptions were thought more likely to report greater changes across the determinants. For example, some people presumably did not perceive the information to be novel because they had been previously exposed to optimistic, biopsychosocial information, and it is not unexpected that these individuals did not feel more confident than before watching the video. To advance our proposed logic, the relationship between perceptions, the video content/delivery features and changes in these determinants needs further exploration. It may be that a ceiling effect occurred in terms of the perceptions, or it may be that some of the strategies used to change the determinants need improving. This may particularly be the case for increasing determinants relating to physical activity behaviour change, as effect sizes obtained for these determinants were smaller. Increased perceived personal relevance of the video was associated with appreciating the importance of physical activity and suggests that efforts to ensure relevance should be maintained and strengthened.

In terms of a direct relationship between participant characteristics and change in behavioural determinants, there was a small and questionable association between higher need for cognition and greater increase in confidence to be physically active after watching the video. Given that confidence to be physically active may be fostered more effectively by this video in those with higher need for cognition, it seems that the video may have catered more in this respect to those people. People with a higher need for cognition tend to enjoy cognitive tasks and process information more elaborately. These people should respond well to a video that presents strong arguments and is factual in terms of intentions and patient activation. People with a lower need for cognition, who rely more on heuristics, emotions and other peripheral cues to process information, were expected to also respond well, given there is sufficient positivity and other peripheral cues to draw from (e.g. university branding suggesting credibility, real people telling their stories) [40, 54]. Systematic reviews suggest that ‘feedback’, ‘action planning’, ‘providing instruction’ and ‘reinforcing effort towards the behaviour’ are the most effective strategies for increasing self-efficacy for physical activity [18, 55], however, these are not easily incorporated into a brief educational video. ‘Vicarious experience’ may also be helpful [55], and this strategy was able to be incorporated into the video. Further exploration of which behaviour change strategies conducive to delivery via educational videos enhance physical activity self-efficacy is important as confidence is considered one of the core predictors of actual behaviour [15].

It is important to highlight that not all educational interventions produced for people with knee OA can be expected to result in similar findings. Effective patient education has been conceptualised as both an ‘art’ and a ‘science’ [56]. Both content and delivery features/strategies are important to consider in developing and evaluating educational interventions. This video included factual content about knee OA (knowledge, information), as well as exploring affective learning (values, beliefs, attitudes) [57], delivered using an empowerment discourse [58]. It included features to cater for both higher and lower need for cognition (although our findings suggest further improvements are recommended for the latter) and positioned to engage people with knee pain as active participants with individual perspectives. The focus of the video was on the main things people can do to manage symptoms and achieve long-term optimal functioning rather than focussing on structural changes and an expectation of symptom worsening. In this respect it is different to typical/traditional education provided to people with the condition. Our findings support this approach as an effective strategy because of the generally positive perceptions and possible improvements in behavioural determinants, which need to be confirmed in experimental studies.

### Limitations

The lack of a comparator group means we cannot know whether the changes in outcome measures can be attributed to the intervention. The responses were measured immediately after watching the video, so sustainability of the changes is also unknown. The improvements in behavioural determinants must therefore be considered with caution. A further limitation is the risk of Type 1 error due to multiple analyses. Generalisability is limited as we inadvertently did not sufficiently capture the views of people with low health literacy. In addition, the sample was limited to people who were exposed to the Australian health services and Australian societal beliefs and attitudes about health and healthcare, and therefore the results may not be the same for people living in different locations/cultures.

## Conclusion

This study adds further support that positively framed video information about knee OA with an emphasis on empowerment for self-managing with physical activity and exercise may be beneficial. Findings are suggestive, though not conclusive, that short term benefits may include improvements in self-efficacy, activation, and motivation/confidence to be physically active. The video investigated in this study appears to be slightly better received by people with higher health literacy and higher need for cognition, as these characteristics were associated with more positive perceptions of the video and greater increase in confidence for physical activity respectively. Further research is needed to confirm these findings, but it is recommended that designers of empowerment education for people with knee OA strive to engage those with lower health literacy and lower need for cognition. Our findings also suggest that education interventions that are enjoyable and perceived to be helpful, and relevant to the individual may have greater effect. Thus, efforts by health professionals to help people with knee OA to perceive the information is being relevant to them personally may optimise the potential for the video to increase motivation and confidence to be physically active.

## Supplementary Information

**Additional file 1.**

## Data Availability

The datasets used and analysed during the current study are available from the corresponding author on reasonable request from thorlene.egerton@unimelb.edu.au.

## Abbreviations

ASES: Arthritis Self-efficacy Scale
BMI: Body mass index
CI: 95% confidence interval
NRS: Numeric rating scale
OA: Osteoarthritis
PAM: Patient Activation Measure
RCT: Randomised controlled trial
SD: Standard deviation
STROBE: STrengthening the Reporting of OBservational studies in Epidemiology
