# Supplementary material for: Exploring changes, and factors associated with changes, in behavioural determinants from a low-cost, scalable education intervention about knee osteoarthritis: An observational cohort study
Source: BMC Musculoskelet Disord. 2021 Oct 9;22:862. doi: 10.1186/s12891-021-04751-2 (PMC8502260; doi:10.1186/s12891-021-04751-2)
Supplement: Supplementary file 1 — Additional file 1. [file 12891_2021_4751_MOESM1_ESM.docx]

Appendix A.

Detailed data collection methods

**Baseline questionnaire**

*Pain severity* was measured using two recommended self-report 11-point numerical rating scales (NRS) [1]: “What is the average amount of pain felt over the past week in your most painful knee?” and “During the past week, what was the worst pain you felt during an activity that usually brings on your knee pain?”. Responses were indicated on a scale from 0 to 10, where 0 is “no pain” and 10 is “your worst pain imaginable”. Scores ranged from 0 to 10 with higher scores indicating worse pain. It is a valid and reliable method to measure pain in this population [2].

*Duration of pain* was categorised as less than one year, 1-2 years, 3-5 years, 5-10 years or more than 10 years.

‘*Need for Cognition*’ is a personality trait relating to the tendency for a person to process information elaborately (e.g. weigh the pros and cons or strengths and weaknesses of an argument) when making decisions. The alternative is to rely on ‘gut feelings’ (e.g. liking it) and heuristics (e.g. ‘a university professor said this, and they are usually right’). ‘Need for Cognition’ was measured using three items and a 11-point NRS. The questions were: 1) I like to have the responsibility of handling a situation that requires a lot of thinking; 2) I would rather do something that requires little thinking/thought than something that is sure to challenge my thinking abilities; and 3) I try to anticipate and avoid situations where there is likely chance that I have to think deeply about something.

*Health literacy* was measured using the 44-item Health Literacy Questionnaire (HLQ); a comprehensive measure of health literacy with established validity [3]. It covers nine conceptually distinct domains: 1) Feeling understood and supported by healthcare providers; 2) Having sufficient information to manage my health, 3) Actively managing my health; 4) Social support for health; 5) Appraisal of health information; 6) Ability to actively engage with healthcare providers; 7) Navigating the healthcare system; 8) Ability to find good health information; and 9) Understand health information well enough to know what to do. The Health Literacy Questionnaire (HLQ) is a comprehensive measure of health literacy developed through a rigorous validity-driven process [3]. Psychometric analyses included confirmatory factor analysis and item response theory.

**Baseline and follow-up questionnaire**

The Arthritis Self-Efficacy Scale (ASES) is a 20-item questionnaire with three subscales that assess self-efficacy for control of pain (5 questions), for physical function (9 questions) and for other arthritis symptoms (6 questions). Questions are rated on 10-point NRSs. The mean value of each of the items in a subscale provides a score for each subscale with higher scores indicating higher levels of perceived self-efficacy. Previous studies support the reliability and validity of this scale for people with osteoarthritis [4].

The Patient Activation Measure (PAM-13) measures attitudes toward self-care [5, 6]. Thirteen statements regarding understanding of health condition and self-management are rated on a 4-point Likert scale. The scale was developed using Rasch analysis and is considered to be valid and reliable [5].

Two NRSs will be used to measure importance and confidence regarding physical activity. The constructs of importance and confidence are based on the Information-Motivation-Behaviour (IMB) behavioural theory [7]. The questions will be: “Please rate how important it is for you to be physically active” and “Please rate how confident you are that you can be sufficiently physically active”, with the anchors of 0=”Not important/confident at all” and 10=”Completely important/confident”. Rating motivation and confidence on 0-10 NRS are commonly used method in behaviour change research and based on recommended for measuring constructs of the Theory of Planned Behaviour (a precursor theory to the IMB) [8].

**Survey**

The feedback survey included 7-point NRSs to measure overall enjoyment, degree of helpfulness, perceived relevance of the information, amount of new information, and perceived credibility of information. A yes/no question asked whether they intended to change any behaviors.

*Perceived relevance* (0-6) was the average of responses to three questions:

1. The information provided was very relevant to me (scored 0 - 6)

2. The information in the video was applicable to my situation (scored 0 - 6)

3. The information in the video seemed like it was written with someone like me in mind (scored 0 - 6)

**References**

[1] G.K. Fitzgerald, R.S. Hinman, J. Zeni, Jr., M.A. Risberg, L. Snyder-Mackler, K.L. Bennell, OARSI Clinical Trials Recommendations: Design and conduct of clinical trials of rehabilitation interventions for osteoarthritis, Osteoarthritis Cartilage 23 (2015) 803-14.

[2] N. Bellamy, Osteoarthritis clinical trials: candidate variables and clinimetric properties, J. Rheumatol. 24 (1997) 768-78.

[3] R.H. Osborne, R.W. Batterham, G.R. Elsworth, M. Hawkins, R. Buchbinder, The grounded psychometric development and initial validation of the Health Literacy Questionnaire (HLQ), BMC Public Health 13 (2013) 658.

[4] K. Lorig, R.L. Chastain, E. Ung, S. Shoor, H.R. Holman, Development and evaluation of a scale to measure perceived self-efficacy in people with arthritis, Arthritis Rheum. 32 (1989) 37-44.

[5] J.H. Hibbard, E.R. Mahoney, J. Stockard, M. Tusler, Development and testing of a short form of the patient activation measure, Health Serv. Res. 40 (2005) 1918-30.

[6] J.H. Hibbard, J. Stockard, E.R. Mahoney, M. Tusler, Development of the Patient Activation Measure (PAM): conceptualizing and measuring activation in patients and consumers, Health Serv. Res. 39 (2004) 1005-26.

[7] W. Fisher, J. Fisher, J. Harman, The information-motivation-behavioral skills model: A general social psychological approach to understanding and promoting health behavior, in: J. Suls, K. Wallston (Eds.), Social psychological foundations of health and illness, Blackwell Publishing, Walden, MA, 2003, pp. 82–106.

[8] I. Ajzen, Constructing a theory of planned behavior questionnaire., <http://people.umass.edu/aizen/index.html>, 2006.
